# Supplementary material for: Neural control of lexical tone production in human laryngeal motor cortex
Source: Nat Commun. 2023 Oct 30;14:6917. doi: 10.1038/s41467-023-42175-9 (PMC10616086; doi:10.1038/s41467-023-42175-9)
Supplement: Supplementary file 1 — Supplementary Information [file 41467_2023_42175_MOESM1_ESM.pdf]

Supplementary Materials

Supplementary Results

Dissociative encoding between tone control and voicing, auditory representation in LMC

Since both laryngeal motor and auditory information were found to be encoded in LMC and laryngeal motor included voicing and tone control here, we next ask what's the relationship between tone control, voicing, and auditory representations. First, we need to find the neural populations that represent tone, voicing and auditory, respectively. Voicing electrodes came from the encoding model, the unique variance explained by binary pitch indicates the degree of voicing encoding. Tone control electrodes are the those with significant *F*-statistics, as we mentioned above. *F*-statistics indicates the degree of tone discriminability. Then we use the auditory responsiveness in high-gamma when the participants (S3-S8) listen to the cue stimulus to define auditory representative electrodes. In other words, these are the electrodes that show a significant listening ERP. We plot the pairwise relationship between the three neural coding populations. There's no strong correlation between auditory representations and tone control (Pearson's $r = 0.03$, $p = 0.38$; Fig. S6a), only a weak correlation between voicing and auditory representations (Pearson's $r = 0.32$, $p = 1.6E\text{-}21$; Fig. S6b) and between tone control and voicing (Pearson's $r = 0.22$, $p = 2.1E\text{-}10$; Fig. S6c). We found a clear spatial dissociation between tone control and auditory representations (Fig. S6d and 6e). The majority of tone control electrodes (84.4%, 27 of 32 tone control electrodes) were not tuned to the auditory stimulus.

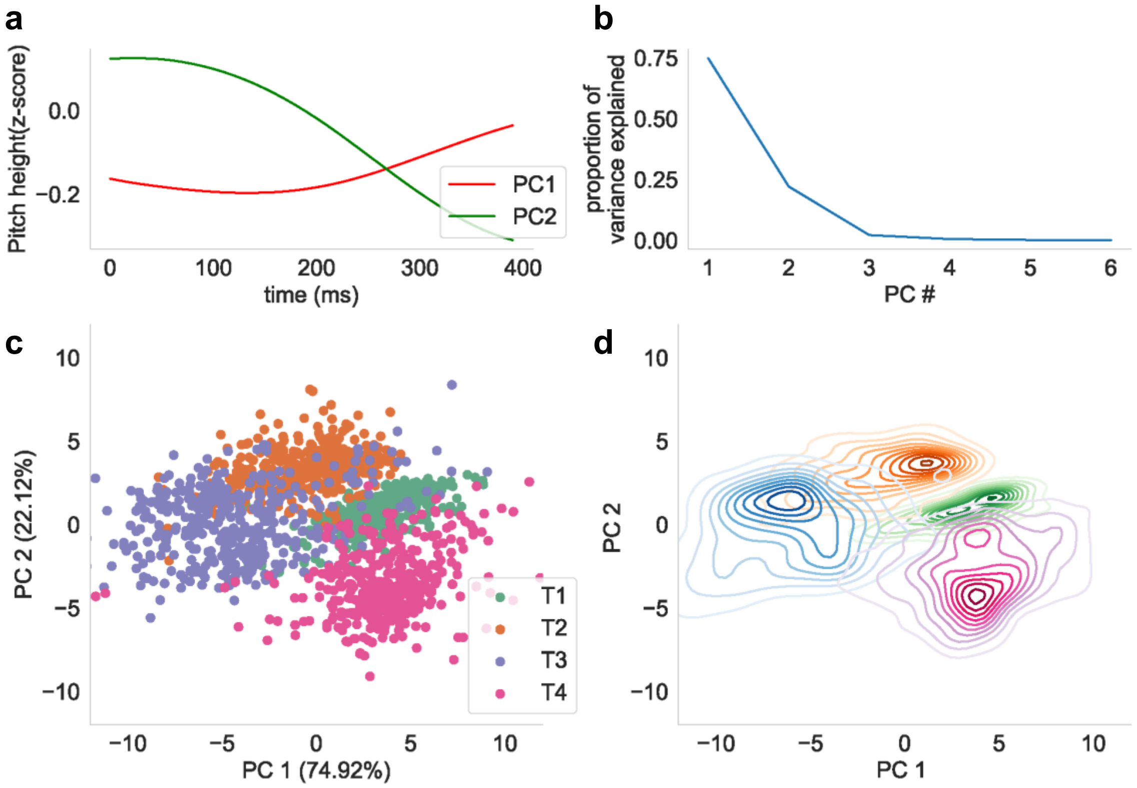

Figure S1. Principal component analysis (PCA) on pitch contour in Mandarin tone production. (a) The time course of the first 2 PCs of the pitch contours from all four lexical tones. **(b)** The accumulated proportion of variance in the pitch contour explained by the first n PCs. **(c)** The scatter plot of pitch contours of the four lexical tones in the space of the first 2 PCs. **(d)** The corresponding probability density of the distributions of the four lexical tones in the PC space.

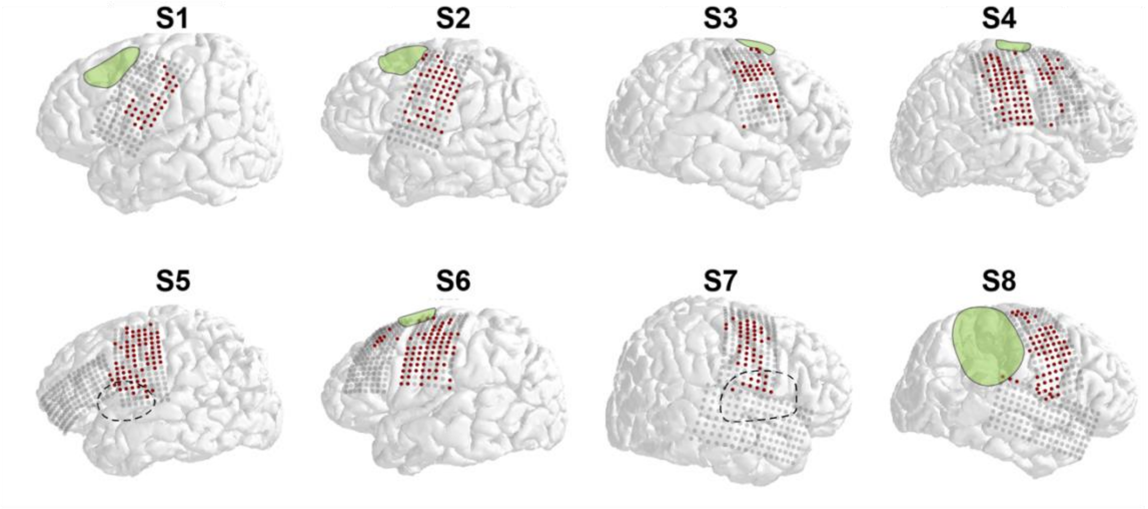

Figure S2. Speech responsive electrodes for all participants (S1-S8). Red dots indicate speech responsive electrodes. Green regions represent the superficial tumor locations. Black dotted lines illustrate the surface projections of the deep insular and medial temporal lobe tumors.

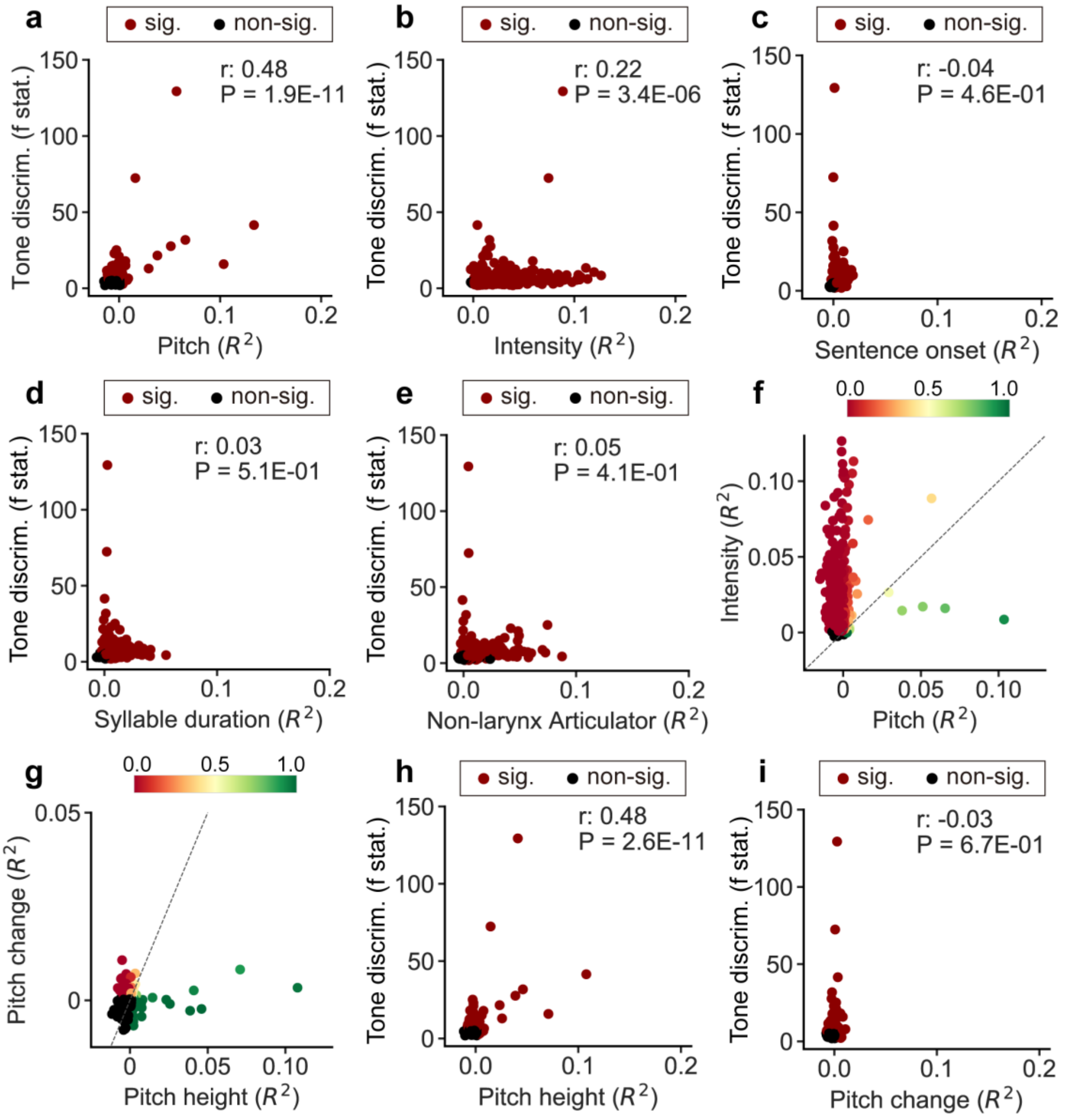

Figure S3. Tone discriminability from neural activity is driven by pitch height representation during natural speech. (a-i) Scatter plot of single electrode encoding properties across speech-selective electrodes from all participants. Each dot represents a single-electrode. The colored dots indicate electrodes that had significant encoding of either x-axis or y-axis encoding features ($p < 0.005$, permutation test), while the black dots indicate non-significant electrodes. The r values are computed among the significant electrodes using Pearson's correlation between the x and y axes. P values are computed using two-tailed t tests. (**a**) Scatterplot of the unique variance explained by pitch features (R^2 of pitch change and pitch height) and tone discriminability (F-statistics across 4 tones). (**b**) Scatterplot of the unique variance explained by intensity (R^2) and tone discriminability (F-statistics). (**c**) Scatterplot of

the unique variance explained by sentence onsets (R^2) and tone discriminability (F-statistics). (**d)** Scatterplot of the unique variance explained by syllable duration (R^2) and tone discriminability (F-statistics). (**e)** Scatterplot of the unique variance explained by non-larynx articulator (R^2) and tone discriminability (F-statistics). **(f)** Scatterplot of the unique variance explained by intensity (R^2) and pitch features (R^2). (**g)** Scatterplot of the unique variance explained by pitch change (R^2) and pitch height (R^2). (**g)** Scatterplot of the unique variance explained by pitch height (R^2) and tone discriminability (F-statistics). (**i)** Scatterplot of the unique variance explained by pitch change (R^2) and tone discriminability (F-statistics).

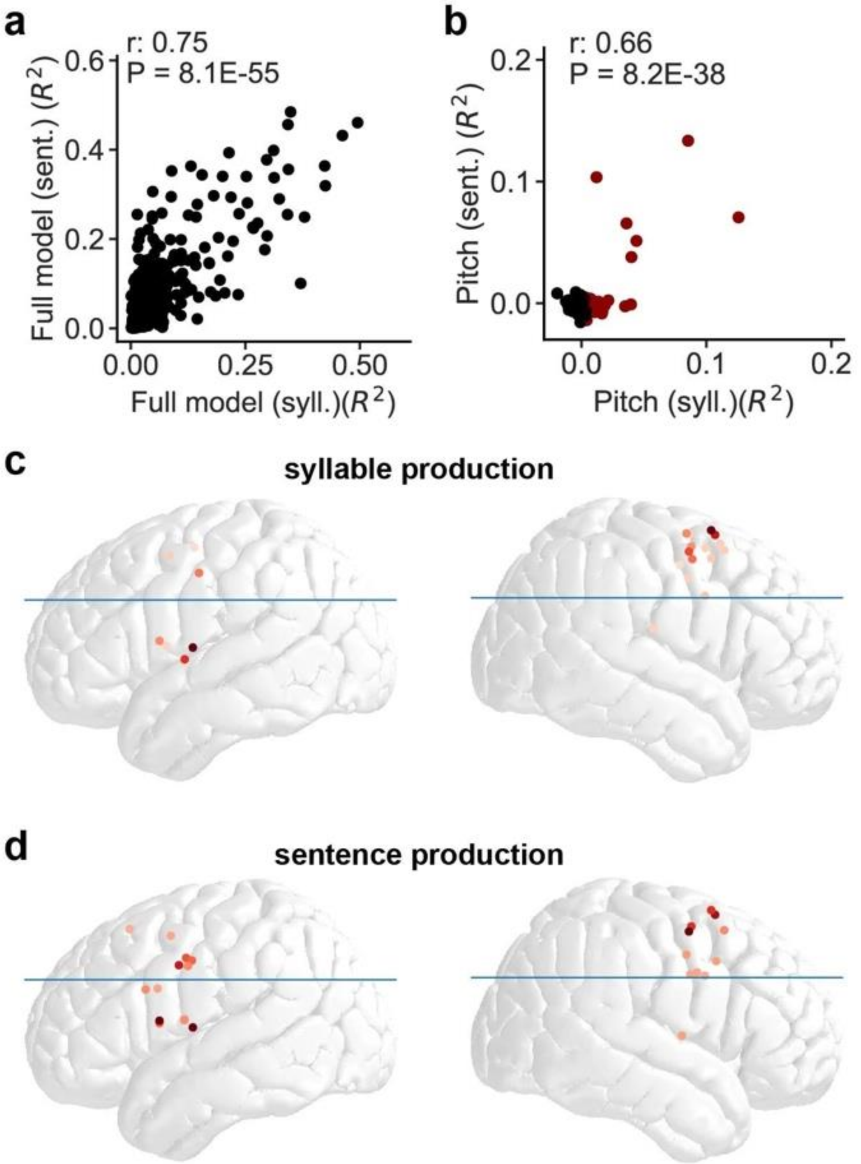

Figure S4. Consistent feature encoding properties and spatial distributions between single syllable word production and sentence production tasks. (a-b) Scatter plot of single electrode encoding properties across speech-selective electrodes from all 5 participants that participated in both syllable and sentence tasks. **(a)** The full model encoding model performance of the syllable production task (full model R^2) versus the sentence production task (full model R^2). **(b)** Scatterplot of the unique variance explained by pitch features in the syllable production task (R^2) and in the sentence production task (R^2). **(c)** The distribution of all tone discriminant electrodes in syllable production task. Color bar indicates the degree of tone discrimination (*F*-statistics across 4 tones). **(d)** The distribution of all tone discriminant electrodes in sentence production task. Color bar indicates the degree of tone discrimination (*F*-statistics across 4 tones).

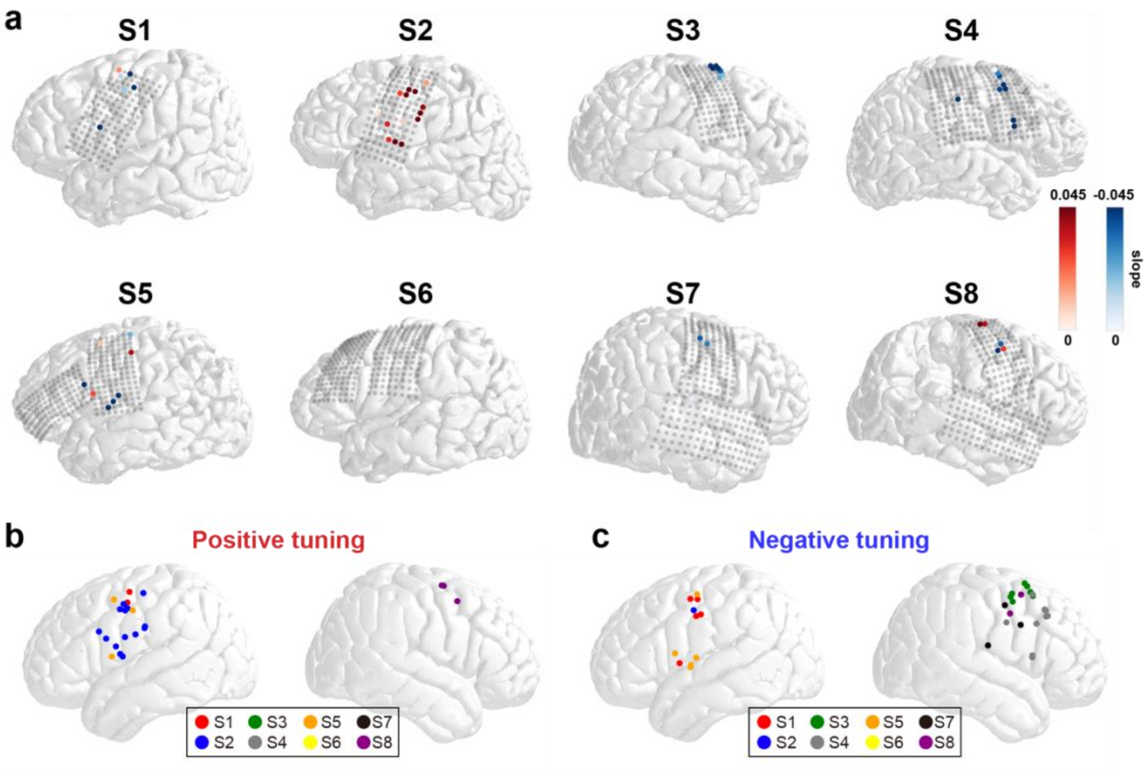

Figure S5. Positive and negative tuning electrodes for all participants (S1-S8), Related to Figure 3. (a) Red dots indicate positive tuning electrodes. Blue dots indicate negative tuning electrodes. Darker color indicates stronger tuning. **(b)** Group-level distribution of positive tuning electrodes on the cortical surface (color coded by subject). **(c)** Group-level distribution of negative tuning electrodes on the cortical surface (color coded by subject).

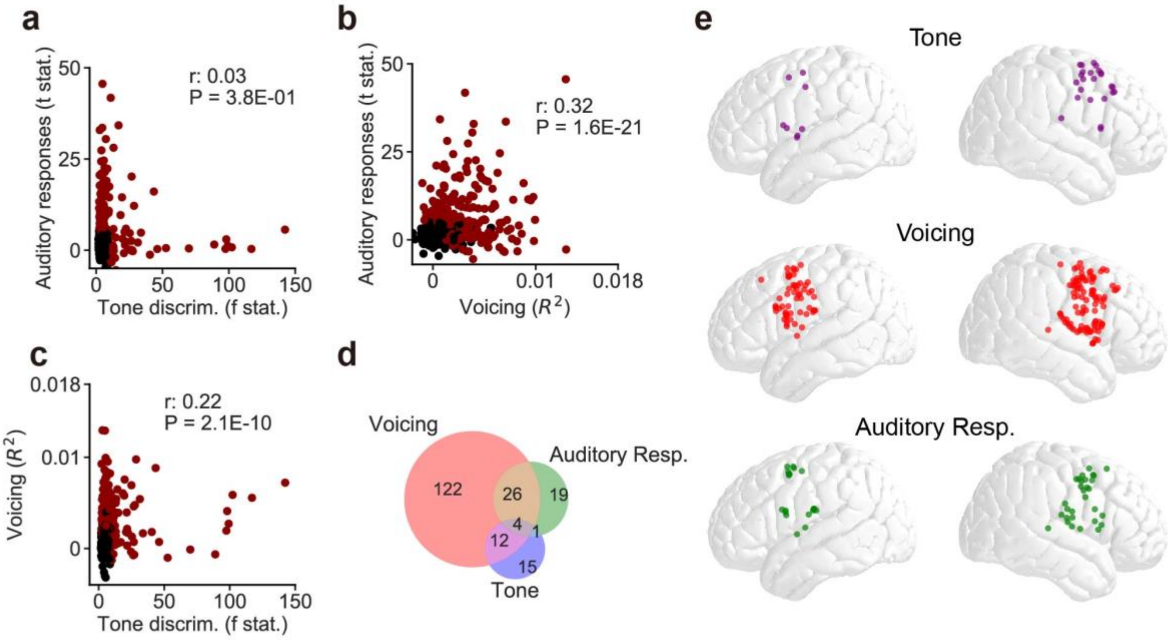

Figure S6. Tone encoding is independent from auditory representation and voicing encoding in vSMC. (a-c) Scatter plot of single electrode encoding properties across speech-selective electrodes from all participants. Each dot represents a single-electrode. The red dots indicate electrodes that had significant encoding of either x-axis or y-axis encoding features ($p < 0.005$, permutation test), while the black dots indicate non-significant electrodes. The *r* values are computed among the significant electrodes using Pearson's correlation between the x and y axes. *P* values are computed using two-tailed *t* tests. (**a**) Scatterplot of the unique variance explained by auditory responses (*t*-statistics) and tone discriminability (*F*-statistics across 4 tones). (**b**) Scatterplot of the unique variance explained by auditory responses (*t*-statistics) and voicing (R^2). (**c**) Scatterplot of the unique variance explained by voicing (R^2) and tone discriminability (*F*-statistics). **(d)** Venn diagram comparing the encoding electrodes of tone control, voicing and auditory responses. **(e)** Bilateral spatial location of encoding electrodes of tone control, voicing and auditory responses across participants S3-S8. Each brain reconstruction shows the normalized scatters illustrating the spatial organization of electrodes on a common brain.
